# The College Council Elections

**Published:** 1921-05-07

**Authors:** Ellen G. Love

**Affiliations:** 14 Sussex Place, Regent's Park, N.W. 1


*To the Editor of* The Hospital.

### The College Council Elections.

A PRIVATE NURSE CANDIDATE.

*To the Editor of* The Hospital.

Sir,—I have been asked to stand as a candidate for election to the Council of the College of Nursing. I am a private nurse, trained at St. Thomas's Hospital, and joined the Red Cross in 1914. I have for some considerable time been a member of the Nurses' Co-operation, 22 Langham Street, and still belong to that Society. Should my fellow-nurses honour me by electing me to the Council, they may rest assured I shall work for their interests in every possible way.—Yours faithfully,

Ellen G. Love.

14 Sussex Place, Regent's Park, N.W. 1, May 2, 1921.

